# Refeeding Syndrome and Its Interventions: A Literature Review

**DOI:** 10.1192/j.eurpsy.2025.1409

**Published:** 2025-08-26

**Authors:** M. A. Rodrigues

**Affiliations:** 1 Universidade Anhembi Morumbi, São Paulo, Brazil

## Abstract

**Introduction:**

Refeeding syndrome is a severe metabolic condition seen in psychiatric patients, particularly those with anorexia nervosa or other eating disorders, after rapid nutrient reintroduction. It involves disturbances such as hypophosphatemia, hypokalemia, and hypomagnesemia, creating challenges in psychiatric and medical settings. Early identification and management are critical to prevent complications like cardiac and respiratory failure (1,2,3).

**Objectives:**

This study analyzes the pathophysiological mechanisms of refeeding syndrome, focusing on key metabolic, psychiatric and electrolyte disturbances during the refeeding process in malnourished patients. It also discusses prevention strategies and clinical management, emphasizing the role of multidisciplinary teams in early diagnosis and treatment (1,2,3).

**Methods:**

A literature review was conducted using Scielo, PubMed, Cochrane, and BMJ, focusing on studies about the pathophysiology, risks, and interventions related to refeeding syndrome. From 40 articles analyzed, 12 published between 2000 and 2023 were selected, focusing on clinical management and treatment guidelines for malnourished patients.

**Results:**

The review highlights that refeeding syndrome (RS) is a serious metabolic condition in malnourished patients, especially those with psychiatric disorders like anorexia nervosa. Rapid nutrient intake can cause metabolic issues, such as hypophosphatemia and hypokalemia, alongside significant psychiatric stress. Anxiety and treatment resistance may increase, especially in patients fearing weight gain, raising the risk of relapse.

Physical discomfort from refeeding, such as fluid retention, can worsen anxiety and complicate treatment. This may lead to extended hospitalization and poor treatment adherence. In psychiatric settings, inadequate management of RS can lead to agitation or self-harm. Preventive measures like controlled carbohydrate intake, thiamine supplementation, and electrolyte monitoring are crucial.

Multidisciplinary teams, including psychiatrists, psychologists, and nutritionists, are key to managing RS. Guidelines like those from NICE recommend gradual refeeding to reduce both metabolic and psychiatric stress.

**Conclusions:**

Refeeding syndrome is a preventable yet potentially fatal condition. Early identification of at-risk patients and careful nutritional strategies are essential to reduce morbidity and mortality. Multidisciplinary teams play a crucial role in managing and educating patients, while further research is needed to inform clinical practices (6-7).

**Disclosure of Interest:**

None Declared

